# Causes of Sterility

**Published:** 1907-04

**Authors:** 


### CAUSES OF STERILITY.

G. G. Ward (Am. Jour. of Obstetrics, August, 1906) has drawn the following conclusions: (1) As conception is dependent upon healthy spermatozoa, normal ova, the union of the same, and the proper implantation of the fertilized egg, so sterility is most frequently dependent upon acquired lesions and congenital defects which cause sterility by interference with these essentials. (2) Many cases of acquired sterility are due to lesions which cause such changes in tubes and ovaries as to prevent the union of spermatozoa and ova. (3) Gonorrhea is the most frequent cause of such lesions. (4) Acquired sterility in many cases is due to endometritis, which causes such changes in the endometrium as to prevent the proper implantation of the impregnated ovum. (5) Sterility associated with flex-

